# Multiple phototherapeutic keratectomy treatments in a Chinese pedigree with corneal dystrophy and an R124L mutation: a 20-year observational study

**DOI:** 10.1186/s12886-019-1167-1

**Published:** 2019-08-22

**Authors:** Li Zeng, Jing Zhao, Yingjun Chen, Jianmin Shang, Aruma Aruma, Xingtao Zhou

**Affiliations:** 1 Department of Ophthalmology, Eye and ENT Hospital of Fudan University, Shanghai, China; 2 NHC Key Laboratory of Myopia (Fudan University), Shanghai, China; 3 Laboratory of Myopia, Chinese Academy of Medical Sciences, Shanghai, China; 4 Research Center of Ophthalmology and Optometry Shanghai, Shanghai, China

**Keywords:** Phototherapeutic keratectomy, Corneal dystrophy, R124L mutation, *TGFBI*

## Abstract

**Background:**

To investigate the efficacy and safety of repeated phototherapeutic keratectomies (PTKs) during long-term treatment for corneal dystrophy (CD) in a Chinese pedigree carrying the R124L mutation in *TGFBI*.

**Methods:**

This was a retrospective review of 20-year medical and genetic records involving five CD patients (10 eyes) from one pedigree. During this period, PTK was conducted for an eye when best-corrected distance visual acuity (BCDVA) reached > 1.0 (LogMAR), due to either primary or recurrent opacities in the cornea. All PTKs were performed by 193-nm excimer laser with or without creation of epithelial flaps. For each eye, routine measurements were conducted for the number of PTKs during follow-up, mean time to recurrence, and BCDVA pre- and post- every PTK (measurements within 3 months from each PTK). Corneal thicknesses measured after the last PTK and at the last visit were analyzed, and subjective satisfaction was assessed.

**Results:**

Gene testing revealed an R124L mutation in *TGFBI*. During 19.60 ± 1.78 years of follow-up, PTKs were conducted twice for three eyes, three times for six eyes, and four times for one eye. After each PTK, effective visual acuity was maintained for 3.60 ± 1.12 years before significant recurrence. BCDVA improved significantly postoperatively than preoperatively for the first PTK for each eye (*p* < 0.001), as well as the second (*p* < 0.001) and third one (*p* < 0.001). After the last PTK and at the final visit, the thinnest corneal thickness was 371.50 ± 56.47 μm and 358.40 ± 101.11 μm, respectively. The average subjective satisfaction score was 8.60 ± 0.89.

**Conclusions:**

Multiple repeated PTKs were effective and safe in a long-term study of CD patients with an R124L mutation in *TGFBI*.

## Background

Corneal dystrophy (CD) is a rare hereditary disease that severely damages visual acuity and is occasionally accompanied by symptoms such as pain, photophobia, and lachrymation, which severely affect patient quality of life. Genetic information is an important factor in diagnosis of corneal dystrophy, and it is now acknowledged that different mutation types of TGFBI gene can lead to different corneal dystrophy subtypes [1]. Surgical treatments such as phototherapeutic keratectomy (PTK) and keratoplasty have been performed for CD patients. However, postoperative redevelopment of opacities in corneas has been frequently reported, especially among patients with some types of CD, such as Reis-Bücklers corneal dystrophy (RBCD) [2–9]. Compared with penetrating keratoplasty (PKP) or lamellar keratoplasty, PTK is less invasive and does not require a corneal graft; thus, it is more efficient for the treatment of CD patients who experience a high rate of recurrence [10]. Previous research demonstrated that, in CD patients treated with PTK, best-corrected visual acuity considerably improved postoperatively [11–15]. Additionally, PTK is effective for treating CD opacities that redevelop postoperatively [16–21]. In patients experiencing a high rate of recurrence, treatment for CD should be performed multiple times. However, follow-up of CD patients treated with multiple PTKs longer than 10 years has rarely been reported. This study reviewed five CD patients from the same family with the R124L mutation of the *TGFBI* gene; all patients underwent multiple PTK treatments over 20 years. We report the outcomes, including safety and efficacy of the procedures, in these patients.

## Materials and methods

This study was performed with approval from the hospital’s institutional review board. Informed consent was obtained from all subjects.

### Study design and participants

This is a retrospective study reviewing medical and genetic records of corneal dystrophy patients who received repeated PTK treatments during approximate 20 year. Inclusion criteria included: 1) patients diagnosed with corneal dystrophy with typical opacities limited in superficial cornea; 2) patients accepted more than 2 times of PTK on one eye. Exclusion criteria included: 1) inflammatory or traumatic ocular diseases; 2) systemic diseases involving corneas; 3) follow up time less than 15 years or significant loss of data. With a focus on the period between 1 January 1996 and 31 December 2017, medical and genetic records of five patients (10 eyes) from a Han Chinese family in Shanghai diagnosed with CD undergoing PTKs were reviewed.

**Fig. 1:**
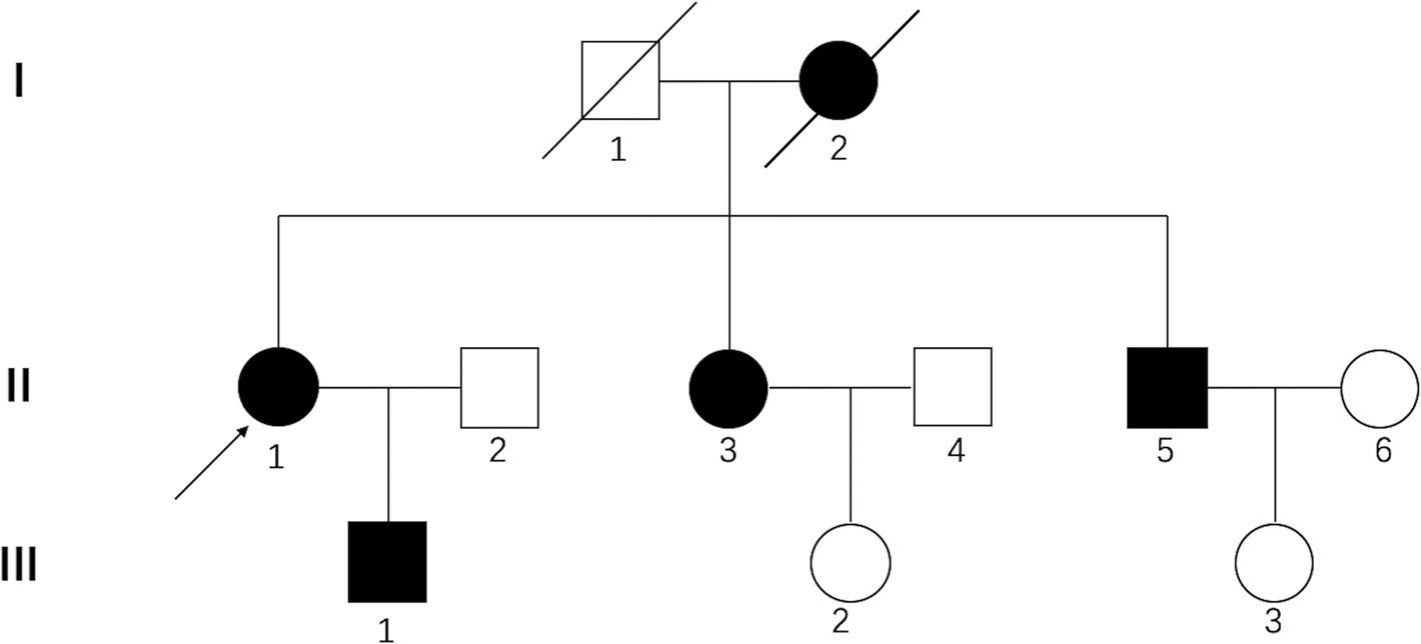
Family tree. I2, II1, II3, II5, and III1 were the 5 patients with corneal dystrophy enrolled in this study

These patients were diagnosed with CD based on impaired vision, occasional pain, and characteristic opacities in corneas under the slit-lamp microscope. Figure 1 shows the family tree. All patients described in this study were identified based on serial numbers in this family tree (I2, II1, II3, II5, and III1); I2 was the proband of this family.

### Genetic analysis

Patients II1 and III1 were tested for the *TGFBI* gene by Sanger sequencing during follow-up. The patients’ whole blood was obtained; anticoagulant tubes were used for storage and transportation of the blood samples. Total RNA extraction of the peripheral blood leukocytes was completed using TRIzol Reagent (Invitrogen, USA) according to the manufacturer’s protocol. Reverse transcription was performed using the Transcriptor First Stand cDNA Synthesis Kit (Roche, SUI). cDNA was amplified by the polymerase chain reaction (PCR) according to standard protocols. All exons of TGFBI were analyzed by Sanger sequencing. The primers were designed as Table 1. The TGFBI reference sequence NM_000358.2 was used.

**Table 1:** Primers used in Sanger tests of TGFBI gene

Prime	Forward	Reverse
TGFBI-1	GTCGCTAGCTCGCTCGGTG	CTCCCAGGGTCTCGTAAAGGTT
TGFBI-2	CTGTCCAGCAGCCCTACCACT	TTGCATGGTGGTCGGCTTT
TGFBI-3	CTAATGGGATTGTAACTGTGAACTG	CGTTGATAGTGAGCATGTCCC
TGFBI-4	GTGTGCTGAAGCCATCGTTG	GGCCAGCTGGTCTTTAATTATGT
TGFBI-5	TCTGTATTCAAAGATGGAACCCCT	AAAGACTGTGTAGACTCCTTCCCG
TGFBI-6	AAGGGAGACAATCGCTTTAGCAT	CTGAGGTCTGTTGGCTGGAGG
TGFBI-7	TCCATGTCATCACCAATGTTCTG	TCTCCCTCCTCCCCCACAT
TGFBIQ	AAGGGAGACAATCGCTTTAGCAT	CCTCCGCTAACCAGGATTTCAT

### PTK procedures, postoperative management, and follow-ups

According to medical records, the indication for PTK treatment was defined as best-corrected distance visual acuity (BCDVA) log of the minimum angle of resolution (LogMAR) > 1.0, due to primary or recurrent opacities during follow-up.

**Fig. 2:**
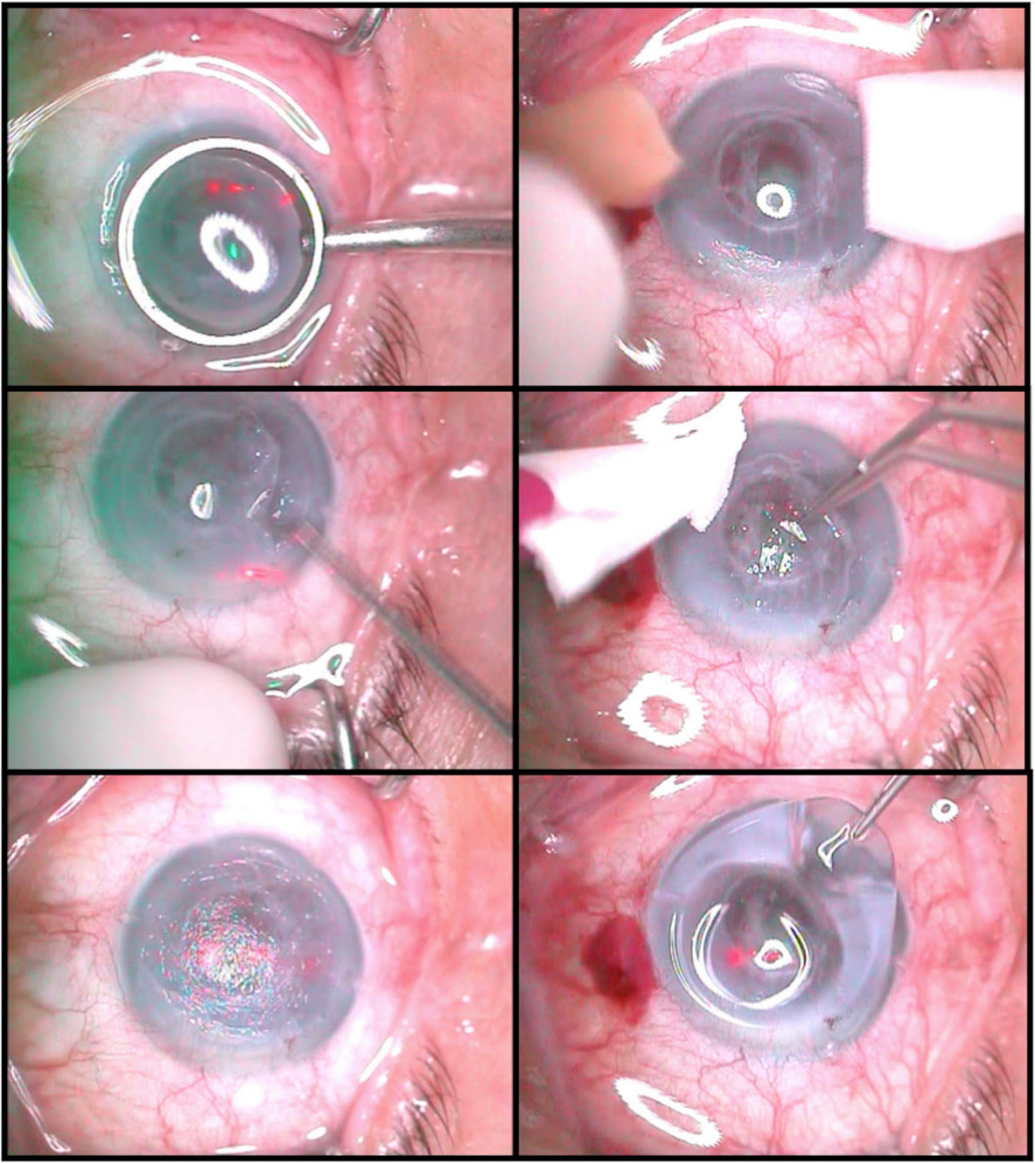
Phototherapeutic keratectomy (PTK) procedures. The images illustrate the subepithelial PTK surgical procedures. Top left: the corneal epithelium was immersed in 20% alcohol. Middle left: the epithelial flap was removed. Bottom left: Bowman’s layer and stroma of the cornea were ablated with laser. Top right: the central zone of the cornea appears transparent after ablation. Middle right: the epithelial flap was repositioned. Bottom right: a bandage contact lens was applied

All surgeries were performed with topical anesthesia. A 193-nm excimer laser was used for all PTKs (Carl Zeiss MEL80; Carl Zeiss, Jena, Germany). The optical zones of all PTKs were set in the range of 5.5–7.0 mm. An approximation of the depth of the corneal deposits was programmed on the computer. Ablation was performed on each eye to a depth of 80–100 μm; if required, an additional ablation of 10–60 μm was applied due to the irregularity of opacities. Alternatively, PTKs without epithelial ablation, but with epithelial flaps, were performed (procedures are presented in Fig. 2).

Bandage contact lenses were applied for 1 week postoperatively. Topical medicines, including antibiotic drops and nonsteroidal anti-inflammatory medication, were applied for 2 weeks. Patients returned regularly until significant recurrence (LogMAR BCDVA > 1.0) required repeated PTK. The final long-term follow-up was performed 3–5 years after the last PTK for each eye.

### Outcome measures and records of recurrence

Routine ophthalmological records of all patients at baseline, before and after each PTK, and at the final visit were reviewed. Slit-lamp microscopy observation, BCDVA, and spectral-domain optical coherence tomography (OCT) (RTVue-100, Optovue; Fremont, CA, USA) results were analyzed. Corneal thickness after the last PTK and at the final visit was reviewed with the Pentacam system (Oculus Optikgeräte; Wetzlar, Germany) and OCT.

The number of PTK treatments for each eye was recorded, and the average time during which effective visual acuity was maintained (TEVAM) after each PTK was calculated. TEVAM was defined as the time required for the patient to experience regression, with an examination result of a LogMAR BCDVA > 1.0 immediately after the last PTK. In particular, to increase the accuracy with respect to preoperative records, if a patient was diagnosed with a LogMAR BCDVA > 1.0 but reported that a long time (> 6 months) had elapsed since he or she began experiencing blurred vision, then the interval time was excluded.

### Subjective satisfaction score

All patients reported the degree of subjective satisfaction regarding the outcomes of the PTKs out of 10 points at the final follow-up. A close caregiver finished scoring for I2. The grading criteria were as follows: 2 points for short-term visual outcome, 2 points for symptomatic improvement, 2 points for improvement of life quality, 2 points for long-term results, and 2 points for acceptability of the recurrence rate. These scores were added to obtain each patient’s total subjective satisfaction score (SSS).

### Statistical analysis

The numbers of PTKs and mean time to recurrence were calculated. Short-term postoperative BCDVA after the first PTK for every eye was compared with preoperative BCDVA by linear mixed model to eliminate the impact of binocular inclusion. The same statistical process was applied to the comparison between short-term postoperative and preoperative BCDVA of the second PTK, as well as the third PTK. Statistical significance was defined as a *p*-value < 0.05. Mean values of long-term BCDVA and corneal thickness, as well as SSS, were calculated. SPSS software 22.0 (IBM Corp.; Armonk, NY, USA) was used to calculate mean values and to perform paired-samples t-tests.

## Results

### Baseline clinical and genetic results

Demographic information, manifestation, and treatment history of all patients at baseline are presented in Table 2. At baseline, all 10 eyes had significant diffuse corneal opacities, uncorrected visual acuity > 1.0, and BCDVA that could not be corrected (all descriptions of visual acuity are presented as LogMAR). Corneal topography findings at baseline exhibited low reliability.

**Table 2:** Basic information of the five subjects at the start of follow-up

Number	Sex	Age	Eye	History of surgical treatment	Baseline BCVA (LogMAR)	Preoperative signs
I2	F	70	OD	PKP once	1.30	All 10 eyes showed diffuse and geographic-like opacities at the level of the Bowman layer and superficial stroma under the slit lamp.
			OS	PKP once	1.30	
II1	F	48	OD	–	1.00	
			OS	–	1.00	
II3	F	46	OD	–	1.30	
			OS	–	1.00	
II5	M	41	OD	–	1.30	
			OS	–	1.00	
III1	M	25	OD	–	1.00	
			OS	–	1.00	

I2, II1, II3, II5, and III1 are serial numbers in the family tree shown in Fig. 1. *BCVA* = best-corrected visual acuity, *F* = female, *M* = male, *PKP* = penetrating keratoplasty. – denotes no history of surgical treatment

**Fig. 3:**
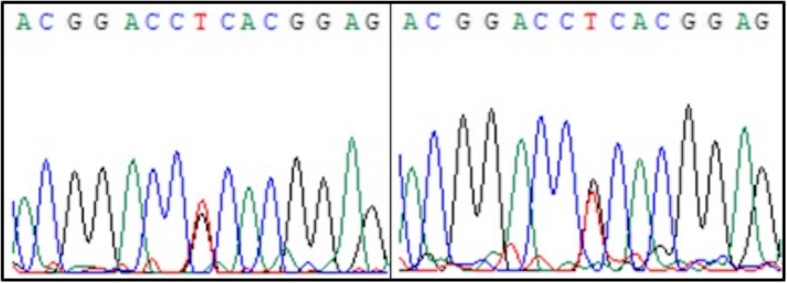
Diagrams of mutant peaks of TGFBI genes of patient II1 and III1. Peaks of c.371G > T mutation of patient II1 (left) and III1 (right) in Sanger sequencing was shown in this picture

The gene test results revealed the presence of a heterozygous R124L mutation (chr5:135382096, c.371G > T, ACGGACC**G**CACGGAG/ACGGACC**T**CACGGAG) in the *TGFBI* gene. Figure 3 showed patients’ diagrams of mutant peaks of TGFBI gene.

### Length of follow-up

The five patients (10 eyes) were followed up for 19.60 ± 1.78 years (range, 16–21 years).

### Amount of PTKs and TEVAM

During the follow-up period (19.60 ± 1.78 years), PTK treatments were conducted twice for three eyes, three times for six eyes, and four times for one eye. In total, 28 PTKs were performed in these five patients. For eight eyes of four patients with no history of PKP before follow-up, the average TEVAM after each PTK was 3.60 ± 1.12 years (*n* = 15). The patient with a history of PKP underwent retreatment with PTK 6 and 7 years after the first PTK for the right and left eyes, respectively.

### Visual and corneal outcomes in the short term

**Fig. 4:**
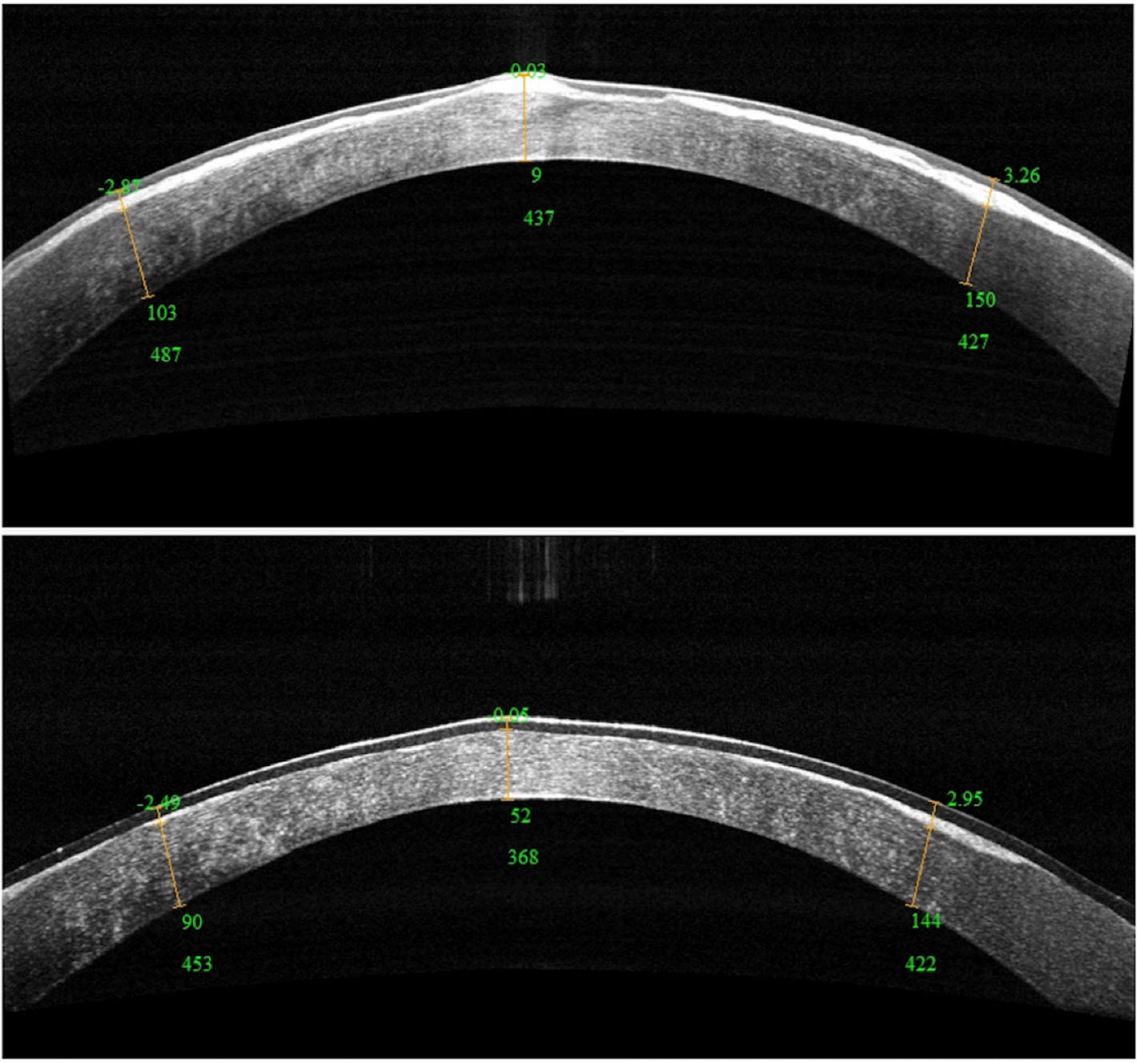
Postoperative optical coherence tomography (OCT). OCT manifestations in the left eye of patient II1 before (top) and 1 day after (bottom) the 3rd phototherapeutic keratectomy (PTK)

Following 28 PTKs during the 20 years of follow-up, postoperative BCDVA improved by three lines or more within 3 months after each PTK. There was a significant difference between the mean postoperative BCDVA and the mean preoperative BCDVA (0.41 ± 0.13 vs 1.20 ± 0.14 LogMAR) for all PTKs (*n* = 28). For the first PTKs (*n* = 10), the second PTKs (n = 10), the third PTKs (*n* = 7), and the fourth PTK (n = 1), the visual outcomes are presented in Table 3. Slit-lamp observation demonstrated reduced opacities and a transparent central zone of the cornea after each PTK within 3 months. Notably, OCT was used for estimation of opacities beginning in the 14th year of follow-up; it showed significant postoperative improvement of corneal transparency (Fig. 4).

**Table 3:** Best corrected distance visual acuity (BCDVA) before and after every phototherapeutic keratectomy

Number of PTK	Preoperative BCDVA (mean value)	Postoperative BCDVA (mean value)	Sample size(n)	Estimate of mean difference	Standard error	*P* value (linear mixed model)
1st	1.12 ± 0.15	0.36 ± 0.07	10	−0.758	0.043	< 0.001
2nd	1.24 ± 0.13	0.44 ± 0.15	10	−0.798	0.052	< 0.001
3rd	1.26 ± 0.11	0.37 ± 0.08	7	−0.883	0.056	< 0.001
4th	1.30	0.40	1	–	–	–

### Long-term visual, corneal, and complicative outcomes

During the follow-up, the final visits of three eyes (three patients) were excluded from the study in the 16th, 19th, and 20th year of follow-up for other surgical treatments, as shown in Table 4. The other five eyes of four patients without PKP history were included in the final follow-up, which took place 3.80 ± 1.10 years after the last PTK. The final follow-up of patient I2 (two eyes), who had history of penetrating keratoplasty (PKP) on both eyes, was conducted in the form of a household survey interview 8 years after the last PTKs due to her Alzheimer’s disease. The visual outcomes of 10 eyes after the last PTKs and at the final visit are presented in Table 4. The household interview of patient I2 showed that she had uncorrected DVA on both eyes of approximately 0.70 (8 years after the last PTKs). The average minimum thickness of the corneas after the last PTKs was 371 ± 56.47 μm after excluding results with low reliability. At the last visit, the average minimum thickness of the cornea was 358.40 ± 101.11 μm. Two eyes ended follow up ahead of time because they received PTK combined with epi-aphakia, which was a new kind of surgery using PTK combing fresh allogeneic corneal stromal lenticule from SMILE to improve patient’s corneal thickness and visual quality while remove of opacities. And the other eye quitted for personal choice of PKP treatment, when repeated PTK and PKP were both alternative treatments at that time. Intraoperative and postoperative complications such as hemorrhage, infection, or corneal ectasia were not observed for any PTK treatment during follow-up. At the end of follow-up, the average SSS was 8.60 ± 0.89.

**Table 4:** Follow-up results for the five subjects

Number	Eye	Number of PTK treatments	Duration of follow-up (years)	3 months after the last PTK		Final follow-up in 2017			
				BCVA (LogMAR)	Thinnest thickness of the cornea (μm)	BCVA (LogMAR)	Thinnest thickness of the cornea (μm)	Subjective scoring	Remarks
I2	OD	2	20	0.70	△	*	*	8	N1
	OS	2	20	0.52	△	*	*		N1
II1	OD	4	21	0.30	△	Hand movement	284	10	Y1
	OS	3	21	0.40	393	0.70	370		Y2
II3	OD	3	16	0.30	380	*	*	8	N2
	OS	3	21	0.30	291	0.11	242		Y3
II5	OD	3	20	0.40	△	*	*	9	N3
	OS	3	21	0.30	△	0.52	395		Y4
III1	OD	2	19	0.30	△	*	*	8	N4
	OS	3	17	0.40	422	0.52	501		Y5
Average	–	2.80 ± 0.63	20	0.39 ± 0.12	371.50 ± 56.47	–	358.40 ± 101.11	8.60 ± 0.89	–

*BCVA* = best-corrected visual acuity, *PKP* = penetrating keratoplasty, *PTK* = phototherapeutic keratectomy

△: Corneal topography results had low reliability. *: Data not available (reasons are represented by N1–4). N1: This follow-up was performed at the patient’s home because of difficulty in mobility. N2–4: These three eyes were not included in the final follow-up because PKP, PTK combined with epi-aphakia, and PTK combined with epi-aphakia were performed in each eye in the 16th (N2), 20th (N3), and 19th (N4) year of follow-up, respectively. Y1–5: the time points of the final follow-up were 5, 3, 3, 3, and 5 years after the last PTK, respectively

## Discussion

The genetic test results in the present study revealed that patients harbored the R124L mutation of TGFBI gene, which is associated with a diagnosis of RBCD [1]. TGFBI gene was first mapped to chromosome 5q31 by Munier et al. [22], and mutation of TGFBI was related with corneal dystrophies. In IC3D, R124L (c371 G > T) mutation of TGFBI gene was indicated as specific to RBDC [1]. And it was illustrated in IC3D edition2: “Regarding mutations of TGFBI, a phenotype typical enough to be clinically recognizable is generally limited to TBCD, RBCD, GCD1, GCD2, and classic LCD. When any of these classic mutations is documented, corresponding terminology should be used without exception, despite any possible individual variation in phenotype.” And on OMIM, the 5 types of mutation of TGFBI gene were also precisely stated that they could cause specific phenotypes of corneal dystrophies respectively: “In addition, the corneal phenotypes caused by changes at R124 and R555 were amino acid specific: R124C in CDL1, R555W and arg124 to ser (R124S; 601692.0008) in CDGG1, R124H in CDA, R124L in CDRB, and R555Q in CDTB.” These summaries helped classification diagnosis based on genetic information.

RBCD caused by R124L mutation is characterized with opacities developed at the level of Bowman layer and superficial stroma, and in some cases opacities extend to the deeper stroma. [1] Corneal dystrophies in epithelium, basal membrane, Bowman’s layer or superficial stromal are indications for PTK [23]. Corneal dystrophies caused by mutation of TGFBI gene was common in superficial cornea, making PTK an appropriate surgical treatment. Patients with R124L mutation of TGFBI experience a high rate of recurrence postoperatively [24]. Repeated PTK treatments appear to be a feasible option for treating recurrent opacities because PTK is minimally invasive and does not require corneal allografts. Although primary PTK for CD improves BCDVA, the efficacy and long-term safety of multiple PTK treatments must be investigated.

Significant improvement in postoperative BCDVA was observed following the multiple PTK procedures performed on patients in this study. Remarkable improvement of postoperative BCDVA was observed after the first PTKs for all eyes (*n* = 10), as well as the second (n = 10) and the third (*n* = 7) PTKs (Table 3). Moreover, PTK was performed four times on one eye; postoperative BCDVA increased significantly after the last operation compared with the preoperative BCDVA. Patients showed satisfaction, as measured by a 10-point system, at the final visit. This indicates that multiple repeated PTK treatments are feasible in patients with R124L mutation and have stable satisfactory outcomes. According to a previous report, patients with mutations in the *TGFBI* gene who had BCDVA improvements of 3.1 lines after PTK did not include those with an R124L mutation [9, 15]. BCDVA was increased by 0.19 ± 0.24 (LogMAR) after PTK in patients with granular CD in another study [13]. A previous study reported that patients with Thiel-Behnke CD diagnosed based on genetic screening had an increase of two lines in postoperative BCDVA compared with preoperative BCDVA [11]. Our study confirmed that PTK is also effective in patients harboring the R124L mutation and further estimated the validity of multiple repeated implements. Notably, the proband of the family (I2) in this study had a history of PKP before follow-up, and each eye of this patient underwent PTK twice during the follow-up period. Improvement in BCDVA was also detected after she underwent PTK. Consistent with previous reports [18], our observations in patient I2 (Fig. 1) suggest that PTK is useful for the treatment of opacities redeveloped after PKP. However, whether and how PKP history influences the efficacy of PTK in patients with CD need to be investigated in future studies.

A previous study on repeated PTK completed 4 years of follow-up [25], and another long-term observational study achieved a similar follow-up period [26]. In our study, the average and maximum follow-up durations for the subjects were 20 and 21 years, respectively. To the best of our knowledge, this study is the first to report such long follow-up durations for patients with CD. Furthermore, three eyes separately underwent PKP, PTK combined with epi-aphakia, and PTK combined with epi-aphakia in the 16th, 19th, and 20th year of follow-up, respectively (Table 4). PTK combined with epi-aphakia was a new kind of surgery using PTK combined with a fresh allogeneic corneal stromal lenticule from SMILE to improve patient’s corneal thickness and visual quality in addition to removement of opacities [27]. Which indicates new surgeries combing PTK might bring new advantages while PTK’s performance in ablation of opacities keep stable. And one eye quitted for personal choice of PKP treatment, when repeated PTK and PKP were both alternative treatments at that time. This indicates that not all patients can completely avoid keratoplasty by multiple PTK treatments. However, our results are consistent with those of a previous study that concluded that PTK had no impact on subsequent PKP treatment [26]; thus, we believe that PTK can harmlessly delay PKP by more than 15 years.

According to previous studies [10, 23], PTK might be accompanied by the risk of delayed corneal epithelial healing or haze. This led us to consider that multiple PTK treatments may pose a higher risk for the abovementioned effects. However, in this study, no complications such as infection, hemorrhage, or corneal ectasia were observed during the follow-up period. Further, BCDVA did not decrease after any treatment, and the thinnest corneal thickness was 371.50 ± 56.47 μm after three PTKs; therefore, we believe that repeated PTKs can be performed safely.

In patients with no history of PKP before follow-up (8 eyes), the average TEVAM after each PTK was 3.60 ± 1.12 years. A previous study reported that 23% of patients with RBCD had significantly redeveloped opacities in the cornea 21.6 months after PTK [6]. Another study focused on patients with different types of CD and showed that redevelopment of opacities after PTK was observed 17.6 months postoperatively [15]. These results slightly differ from those of our study, which may be due to differing genetic bases. However, various definitions of recurrence in the literature should be considered as a factor. Whether different gene types influence the time for which corneas maintain transparency after PTK must be further investigated. The patient (I2) who had PKP history before the start of follow-up underwent a second PTK in both eyes 6 and 7 years after the first PTK, respectively. According to previous studies, the recurrence rate of granular corneal dystrophies 10 years after PKP was 43% [8]. Another study reported that mild recurrence was observed at a mean of 32 and 47 months after PTK, and significant recurrence was observed at a mean of 36 and 50 months, after PTK over a previous full-thickness graft [16], which is similar to the outcome of our study. Our results reveal that the recurrence of opacities is inevitable after PTK for corneal grafts. However, future cohort studies are needed to confirm whether there is a significant difference in the time for recurrence between patients with and without history of PKP.

This study analyzed 20-year records of PTKs repeatedly performed for CD patients with the R124L mutation in the *TGFBI* gene in order to illustrate the efficacy and safety of multiple PTKs on a single cornea. However, the sample size was small and the 20-year data were not integrated. After uncovering of association between different mutations of TGFBI gene with significant phenotypes of corneal dystrophies, it is feasible to explore the outcome of PTK treating corneal dystrophies with different TGFBI gene types. A cohort study with a large sample size in the future is necessary for more accurate evaluation of multiple PTKs over an extended period of time, certainty based on more comprehensive genetic diagnosis and more accurate clinical estimations. In large-sample cohort studies, factors like region, environment and history of PKP are worth comparison.

## Conclusion

In conclusion, multiple repeated PTKs had long-term efficacy and safety in treating CD patients with an R124L mutation in *TGFBI*, with or without history of PKP.

## Data Availability

The datasets of the current study are available upon request from the co-first authors Li Zeng and Jing Zhao.

## Abbreviations

BCDVA: Best-corrected distance visual acuity
CD: Corneal dystrophy
LogMAR: Log of the minimum angle of resolution
OCT: Optical coherence tomography
PKP: Penetrating keratoplasty
PTK: Phototherapeutic keratectomy
RBCD: Reis-Bücklers corneal dystrophy
SSS: Subjective satisfaction score
TEVAM: average time during which effective visual acuity was maintained

## References

[1] Weiss JS, Moller HU, Aldave AJ, Seitz B, Bredrup C, Kivela T, Munier FL, Rapuano CJ, Nischal KK, Kim EK (2015). IC3D classification of corneal dystrophies-edition 2. Cornea.

[2] Cheng J, Qi X, Zhao J, Zhai H, Xie L (2013). Comparison of penetrating Keratoplasty and deep lamellar Keratoplasty for macular corneal dystrophy and risk factors of recurrence. Ophthalmology.

[3] Inoue T, Watanabe H, Yamamoto S, Inoue Y, Okada M, Hori Y, Maeda N, Inoue Y, Hayashi K, Shimomura Y, Tano Y (2001). Different recurrence patterns after phototherapeutic keratectomy in the corneal dystrophy resulting from homozygous and heterozygous R124H BIG-H3 mutation. Am J Ophthalmol.

[4] De la Paz M, Barraquer RI, Alvarez De Toledo JP, Buigues A, Michael R, Barraquer JI. Recurrence of anterior corneal dystrophies after keratoplasty. Acta Ophthalmol. 2013;91.

[5] Sorour HM, Yee SB, Peterson NJ, Li FT, Macsai MS, Zhao XC, Yee RW (2005). Recurrence of chromosome 10 Thiel-Behnke corneal dystrophy (CDB2) after excimer laser phototherapeutic keratectomy or penetrating keratoplasty. Cornea.

[6] Dinh R, Rapuano CJ, Cohen EJ, Laibson PR (1999). Recurrence of corneal dystrophy after excimer laser phototherapeutic keratectomy. Ophthalmology.

[7] Inoue T, Watanabe H, Yamamoto S, Maeda N, Inoue Y, Shimomura Y, Tano Y (2002). Recurrence of corneal dystrophy resulting from an R124H big-h3 mutation after phototherapeutic keratectomy. Cornea.

[8] Marcon AS, Cohen EJ, Rapuano CJ, Laibson PR (2003). Recurrence of corneal stromal dystrophies after penetrating keratoplasty. Cornea.

[9] Lewis DR, Price MO, Feng MT, Price FW (2017). Recurrence of granular corneal dystrophy type 1 after phototherapeutic keratectomy, lamellar Keratoplasty, and penetrating Keratoplasty in a single population. Cornea.

[10] Rathi VM, Vyas SP, Sangwan VS (2012). Phototherapeutic keratectomy. Indian J Ophthalmol.

[11] Dogru M, Katakami C, Nishida T, Yamanaka A (2001). Alteration of the ocular surface with recurrence of granular/Avellino corneal dystrophy after phototherapeutic keratectomy: report of five cases and literature review. Ophthalmology.

[12] Hieda O, Kawasaki S, Wakimasu K, Yamasaki K, Inatomi T, Kinoshita S (2013). Clinical outcomes of phototherapeutic keratectomy in eyes with Thiel-Behnke corneal dystrophy. Am J Ophthalmol.

[13] Das S, Langenbucher A, Seitz B (2005). Excimer laser phototherapeutic keratectomy for granular and lattice corneal dystrophy: a comparative study. J Refract Surg.

[14] Seitz B, Behrens A, Fischer M, Langenbucher A, Naumann GOH (2004). Morphometric analysis of deposits in granular and lattice corneal dystrophy - histopathologic implications for phototherapeutic keratectomy. Cornea.

[15] Gruenauer-Kloevekorn C, Braeutigam S, Froster UG, Duncker GIW (2009). Surgical outcome after phototherapeutic keratectomy in patients with TGFBI-linked corneal dystrophies in relation to molecular genetic findings. Graefes Arch Clin Exp Ophthalmol.

[16] Reddy JC, Rapuano CJ, Nagra PK, Hammersmith KM (2013). Excimer laser phototherapeutic keratectomy in eyes with corneal stromal dystrophies with and without a corneal graft. Am J Ophthalmol.

[17] Hafner AU, Seitz B, Langenbucher A, Naumann GOH: Phototherapeutic keratectomy (o-PTK) for recurrent granular and lattice dystrophy after corneal transplantation using the 193 nm excimer laser- long-term results of 20 consecutive procedures. Invest Ophthalmol Vis Sci 2003, 44:U336-U336.

[18] Ellies P, Bejjani RA, Bourges JL, Boelle PY, Renard G, Dighiero P (2003). Phototherapeutic keratectomy for BIGH3-linked corneal dystrophy recurring after penetrating keratoplasty. Ophthalmology.

[19] Rathi VM, Taneja M, Murthy SI, Bagga B, Vaddavalli PK, Sangwan VS (2016). Phototherapeutic keratectomy for recurrent granular dystrophy in postpenetrating keratoplasty eyes. Indian J Ophthalmol.

[20] Cavanaugh TB, Lind DM, Cutarelli PE, Mack RJS, Durrie DS, Hassanein KM, Graham CE (1999). Phototherapeutic keratectomy for recurrent erosion syndrome in anterior basement membrane dystrophy. Ophthalmology.

[21] Sridhar MS, Rapuano CJ, Cosar CB, Cohen EJ, Laibson PR (2002). Phototherapeutic keratectomy versus diamond burr polishing of Bowman's membrane in the treatment of recurrent corneal erosions associated with anterior basement membrane dystrophy. Ophthalmology.

[22] Munier FL, Korvatska E, Djemai A, Le Paslier D, Zografos L, Pescia G, Schorderet DF (1997). Kerato-epithelin mutations in four 5q31-linked corneal dystrophies. Nature Genet.

[23] Fagerholm P (2003). Phototherapeutic keratectomy: 12 years of experience. Acta Ophthalmol Scand.

[24] Stewart OG, Pararajasegaram P, Cazabon J, Morrell AJ (2002). Visual and symptomatic outcome of excimer phototherapeutic keratectomy (PTK) for corneal dystrophies. Eye.

[25] Nassaralla BA, Garbus J, McDonnell PJ (1996). Phototherapeutic keratectomy for granular and lattice corneal dystrophies at 1.5 to 4 years. J Refract Surg.

[26] Szentmary N, Langenbucher A, Hafner A, Seitz B (2004). Impact of phototherapeutic keratectomy on the outcome of subsequent penetrating keratoplasty in patients with stromal corneal dystrophies. Am J Ophthalmol.

[27] Zhao J, Sun L, Shen Y, Tian M, Yao P, Zhou X (2016). Using donor Lenticules obtained through SMILE for an Epikeratophakia technique combined with phototherapeutic keratectomy. J Refract Surg.

